# Altered norepinephrine transmission after spatial learning impairs sleep-mediated memory consolidation in rats

**DOI:** 10.1038/s41598-023-31308-1

**Published:** 2023-03-14

**Authors:** Ernesto Durán, Martina Pandinelli, Nikos K. Logothetis, Oxana Eschenko

**Affiliations:** 1 Department of Physiology of Cognitive Processes, Max Planck Institute for Biological Cybernetics, 72076 Tübingen, Germany; 2 International Center for Primate Brain Research, Center for Excellence in Brain Science and Intelligence Technology (CEBSIT), Institute of Neuroscience (ION), Chinese Academy of Sciences, Shanghai, China; 3 Division of Imaging Science and Biomedical Engineering, University of Manchester, Manchester, M13 9PT UK

**Keywords:** Neuroscience, Learning and memory, Consolidation

## Abstract

The therapeutic use of noradrenergic drugs makes the evaluation of their effects on cognition of high priority. Norepinephrine (NE) is an important neuromodulator for a variety of cognitive processes and may importantly contribute to sleep-mediated memory consolidation. The NE transmission fluctuates with the behavioral and/or brain state and influences associated neural activity. Here, we assessed the effects of altered NE transmission after learning of a hippocampal-dependent task on neural activity and spatial memory in adult male rats. We administered clonidine (0.05 mg/kg, i.p.; n = 12 rats) or propranolol (10 mg/kg, i.p.; n = 11) after each of seven daily learning sessions on an 8-arm radial maze. Compared to the saline group (n = 9), the drug-treated rats showed lower learning rates. To assess the effects of drugs on cortical and hippocampal activity, we recorded prefrontal EEG and local field potentials from the CA1 subfield of the dorsal hippocampus for 2 h after each learning session or drug administration. Both drugs significantly reduced the number of hippocampal ripples for at least 2 h. An EEG-based sleep scoring revealed that clonidine made the sleep onset faster while prolonging quiet wakefulness. Propranolol increased active wakefulness at the expense of non-rapid eye movement (NREM) sleep. Clonidine reduced the occurrence of slow oscillations (SO) and sleep spindles during NREM sleep and altered the temporal coupling between SO and sleep spindles. Thus, pharmacological alteration of NE transmission produced a suboptimal brain state for memory consolidation. Our results suggest that the post-learning NE contributes to the efficiency of hippocampal-cortical communication underlying memory consolidation.

## Introduction

Clinical use of noradrenergic drugs makes evaluation of their effects on cognitive functions of high importance^1,2^. Clonidine and propranolol are among the most commonly prescribed drugs. Clonidine is used as an antihypertensive and analgetic but is also prescribed for the medication of sleep disturbances, and since recently for treatment of attention deficit hyperactivity disorder (ADHD) and Parkinson’s disease^1,3,4^. Propranolol is used for the treatment of several cardiovascular conditions and anxiety^5^. Despite the obvious health benefits, the pharmacological alteration of norepinephrine (NE) levels may, as a side effect, impair patients' cognitive abilities. The essential role of NE transmission for conscious (or ‘online’) information processing is well-established^2,6,7^. Earlier studies have emphasized the importance of NE also during unconscious (or ‘offline’) states like sleep^8–10^, when the learning-induced molecular, synaptic, and circuit modifications take place^11^. During post-learning sleep, initially labile memory traces stabilize via reactivation of memory-encoding neural representations in the hippocampus (HPC) and are subsequently integrated into the neocortex for long-term storage^12^. It has been shown that pharmacological alternation of NE level after learning affected memory consolidation in animals^13–18^ and humans^9,10,19,20^. Furthermore, the behavioral pharmacology studies are consistent with a facilitatory role of NE for synaptic plasticity^21–25^. Despite extensive evidence provided by psychopharmacology on the role of NE for memory encoding and consolidation, the post-learning dynamics of NE transmission has not been systematically characterized. Recent studies by monitoring NE release at a high temporal resolution in spontaneously behaving mice have further refined earlier work and proposed a role of NE release for the continuity of non-rapid eye movement (NREM) sleep and NREM sleep-mediated memory consolidation^26,27^.

The neocortical slow (~ 1 Hz) oscillations (SO), thalamocortical sleep spindles (10–16 Hz), and hippocampal ripples (~ 150 Hz) are the hallmarks of NREM sleep and have been identified as the key oscillations enabling sleep-dependent systems-level memory consolidation^28^. The impact of NE in temporally coordinated activity within a memory-supporting cortico-hippocampal network remains insufficiently understood^11^. Microdialysis measurement of the prefrontal NE release in the rat detected a transient increase ~ 2 h after odor discrimination while blocking the prefrontal β-adrenoreceptors after learning caused memory deficit^29^. In the dorsal HPC, activation of β-adrenoreceptors is required for the efficient consolidation of emotional and spatial memory^30,31^. Both, α1- and β-adrenergic receptors are involved in the generation of sleep spindles and hippocampal ripples^26,32,33^. Previously, we have documented elevated activity of the Locus Coeruleus (LC) noradrenergic neurons during NREM sleep after learning^34^ and their coordinated firing with SO^35^. In our recent study, we showed that in anesthetized rats the LC phasic activation modulated cortical population dynamics at the temporal scale of a single SO cycle^36^. Finally, alteration of naturalistic LC activity patterns during NREM sleep led to memory deficit^37,38^.

In the present study, we linked a deficiency of spatial learning with an altered neural activity within the memory-supporting network. To alter NE transmission, we used clonidine and propranolol, two clinically relevant drugs. Clonidine suppresses the firing of the LC-NE neurons via activation of α2-adrenoreceptors and reduces NE release from the LC terminals^39^. Propranolol blocks the NE transmission by binding to β-adrenoreceptors and after repeated treatment at a moderate dose (< 40 mg/kg) inhibits the LC activity^40,41^. We report that the alteration of NE transmission during the post-acquisition period impaired spatial learning. The reduction of SO, sleep spindles, and hippocampal ripples together with the appearance of high voltage spike-and-wave patterns (HVS) was indicative of a pharmacologically induced switch to a brain state that was likely suboptimal for memory consolidation. Our results further support the view that NE transmission during NREM sleep importantly contributes to information processing within the thalamocortical and hippocampal-cortical networks, which are thought to mediate systems-level memory consolidation.

## Results

The results were obtained from the adult (300—400 g) male Sprague–Dawley rats (n = 38) split randomly into three drug conditions (clonidine, propranolol, and saline). The conditions for each rat and the data inclusion/exclusion are listed in Supplementary Table S1.

### Post-learning clonidine and propranolol cause spatial memory deficit

**Figure 1:**
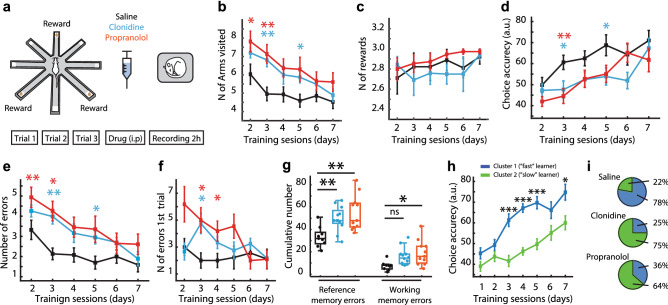
Effects of clonidine and propranolol on consolidation of spatial memory. (**a**) The spatial learning task and experimental design. Rats were allowed to collect rewards from three fixed locations on an 8-arm radial maze during three daily trials for seven consecutive days. After each training session, rats received a systemic drug injection and their behavior and neural activity were monitored for 2 h. (**b**–**e**) The behavioral variables averaged over 3 daily trials are shown for different experimental groups. (**b**) Number of entered maze arms; (**c**) number of rewards; (**d**) choice accuracy; (**e**) number of errors. (**f**) The number of errors during the first trial are shown for different experimental groups. (**g**) The cumulative number of reference and working memory errors for different experimental groups. (**h**) The between-subject differences in the learning dynamics. The K-means cluster analysis revealed fast and slow learners. (**i**) The proportion of fast and slow learners for different experimental groups. Box-whisker plot shows the median, the first and third quartiles, min/max and the outliers; dots show individual datapoints. *p < .05; **p < .01 (Bonferroni post-hoc comparisons vs. saline).

We trained a total of 32 rats on a spatial memory task during seven daily sessions (Fig. 1a; see Supplementary Methods for details). Twenty-six rats were implanted with chronic electrodes for monitoring the frontal EEG and local field potentials (LFP) in the hippocampus (see Methods for details). Additional 6 rats were intact (non-implanted) but followed the same training/drug protocol. Importantly, there was no difference in the learning rate between the implanted and non-implanted rats (Supplementary Figure S1). Immediately after each learning session rats received clonidine (0.05 mg/kg, i.p.; n = 12), propranolol (10 mg/kg, i.p.; n = 11), or saline (1 ml/kg, i.p.; n = 9). In the first (drug-free) learning session, there was no between-group difference by any behavioral variable (Supplementary Table S1). In the course of learning, all rats actively entered different maze arms (Fig. 1b) and collected all rewards almost in every trial (Fig. 1c); this behavioral pattern indicated no between-group difference in general locomotor activity or food motivation. Most behavioral variables reflected the learning dynamics. The accuracy of task performance was gradually increasing (repeated measures ANOVA, F (3.7,106.6) = 12.0, *p* < 0.0001; Fig. 1d) with the between-group difference approaching a significance level (F (2,29) = 3.33, *p* = 0.05). The overall number of errors was significantly higher in the drug-treated rats (Fig. 1e). There was a significant effect of repetition (F (5,145) = 11.0, *p* < 0.001) and between-group difference by the number of errors (F(2,29) = 7.34, *p* = 0.003). The post-hoc comparisons confirmed a higher number of errors in the drug-treated groups (clonidine: *p* = 0.031, propranolol: *p* = 0.002; Bonferroni post-hoc comparisons vs. saline). Notably, propranolol-treated rats made the most errors during the first trial (Fig. 1f); the latter was indicative of a memory retrieval deficit. To further explore the nature of spatial memory deficit, we split the errors into working memory (WM, re-entering the maze arm within a trial) and reference memory (RM, entering unbaited maze arm) errors. There was a significant between-group difference by both WM (F (2, 29) = 8.0, *p* = 0.002) and RM (F (2, 29) = 5.0, *p* = 0.014). The post-hoc comparisons showed that drug-treated rats committed significantly more RM errors (Fig. 1g). Besides, the propranolol-treated rats also made more WM errors (Fig. 1g). Despite obvious spatial memory deficit at the early stages of learning, all rats reached a similar performance level by the end of the learning period (Fig. 1d,e). The trial time was decreasing (F (3.5, 102.4) = 16.1, *p* < 0.0001), yet equally in all groups (time: F (2,29) = 0.43, *p* = 656). Finally, we used the K-means cluster analysis to split rats according to their learning speed (see Methods for details). Indeed, individual rats learned the task at different rates (Fig. 1h). Remarkably, the proportion of ‘slow’ learners was much higher among the drug-treated rats (Fig. 1i).

### Clonidine and propranolol suppress hippocampal ripples

**Figure 2:**
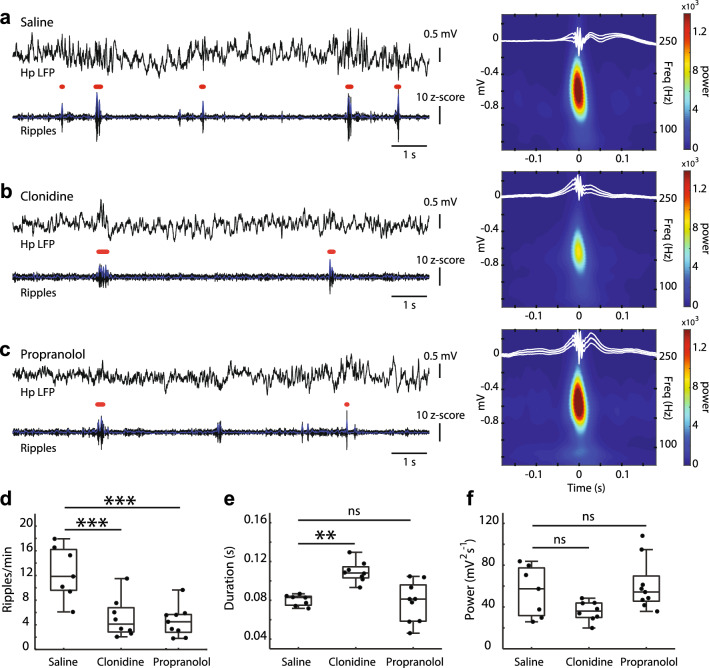
Modulation of the hippocampal ripples by clonidine and propranolol. (**a**–**c**) Representative traces of the hippocampal local field potentials (LFP, 0.1–1 kHz) and corresponding band-pass (120–250 Hz) filtered LFP showing ripple oscillations under different drug conditions. Horizontal red bars above the filtered traces show detected ripples. The right panels show the group-averaged peri-event spectrograms and the wave averages (± SEM) of the hippocampal LFP around the ripple peak. (**d**–**f**) The effects of drugs on the ripple rate (**d**), duration (**e**), and power (**f**). All detected ripples in all behavioural states were included for analysis (saline: 728.0 ± 100.3 ripples/rat; clonidine: 310.0 ± 67.0 ripples/rat; propranolol: 284.3 ± 50.1 ripples/rat). Box-whisker plots show the median, the 1st and 3rd quartiles, min/max, and the outliers; dots show individual datapoints. *p < .05; ***p < .001 (Bonferroni post-hoc comparisons vs. saline).

We assessed the effects of clonidine and propranolol on the hippocampal ripples as the key elements of offline memory consolidation^12^. Retrospective analysis revealed that in 14 out of 26 rats, which were initially implanted and trained on the maze, the ripple detection was not reliable due to various technical failures. Therefore, to increase the sample size, the EEG and hippocampal LFPs were recorded from additional 6 rats, which were treated with drugs, but not tested on the maze (Supplementary Figure S1). Thus, the effects of drugs on the hippocampal ripples were tested in 24 rats (clonidine, n = 8; propranolol, n = 9, and saline, n = 7; Supplementary Table S1). Both drugs significantly reduced the ripple rate for at least 2 h after injection (F(2, 23) = 13.27, *p* = 0.0002; Fig. 2a–d). Classifying rat behavior into active wakefulness (AW), quiet wakefulness (QW), and NREM sleep (see Methods for details) revealed that the most pronounced effect of both drugs occurred during NREM sleep compared to AW and QW episodes (Table 1). Namely, compared to the saline group (n = 7), the degree of ripple suppression during NREM sleep varied from 40.1% to 91.3% after clonidine (n = 8) and from 2.3% to 86.6% after propranolol (n = 9) injection. Apart from a striking decrease in the ripple rate, no drug effects were revealed on the ripple intrinsic properties (amplitude: F(2, 23) = 1.12, *p* = 0.34; power: F(2, 23) = 3.3, *p* = 0.056; intra-ripple frequency: (F(2, 23) = 2.40, *p* = 0.11), except for a longer ripple duration after clonidine (F(2, 23) = 11.47, *p* = 0.0004; Fig. 2d–f).

**Table 1:** The rate of oscillatory events for different behavioral states and groups.

Event	Drug	Active wakefulness	Quiet wakefulness	NREM sleep
Ripples, events/min	*Saline*	5.2 ± 0.6	13.1 ± 2.5	17.9 ± 2.6
	*Clonidine*	4.3 ± 1.2	5.5 ± 1.4**⇓	5.4 ± 1.2***⇓
	*Propranolol*	1.9 ± 0.5*⇓	5.9 ± 0.7**⇓	8.1 ± 1.6**⇓
SO, events/min	*Saline*	1.1 ± 0.2	2.7 ± 0.6	16.8 ± 0.4
	*Clonidine*	2.3 ± 0.6	2.5 ± 0.5	6.5 ± 2.0***⇓
	*Propranolol*	1.1 ± 0.3	2.7 ± 0.6	17.5 ± 1.2
Spindles, events/min	*Saline*	0.1 ± 0.03	0.2 ± 0.09	5.9 ± 0.1
	*Clonidine*	1.5 ± 0.5**⇑	2.2 ± 0.6**⇑	4.5 ± 0.3**⇓
	*Propranolol*	0.2 ± 0.1	0.6 ± 0.2	5.7 ± 0.2
HVS, events/min	*Saline*	0.002 ± 0.002	0.04 ± 0.03	0.2 ± 0.01
	*Clonidine*	0.1 ± 0.05	0.2 ± 0.06	0.4 ± 0.05***⇑
	*Propranolol*	0.05 ± 0.03	0.07 ± 0.02	0.2 ± 0.01

Means ± S.E.M. and the range (min/max) for each variable are shown. *p < 0.05, **p < 0.01, and ***p < 0.001 for comparisons vs. saline (Bonferroni post-hoc test). Arrows indicate a significant increase (⇑) or decrease (⇓).

### Effects of clonidine and propranolol on the behavioral state and sleep-associated EEG oscillations

After the exclusion of invalid EEG data (n = 5 rats), the EEG-based analyses were performed using a total of 21 datasets (clonidine, n = 6; propranolol, n = 9, and saline, n = 6; Supplementary Table S1). In addition to the effects on hippocampal population activity, both drugs affected the pattern of spontaneous behavior. There was a between-group difference in overall time spent in each behavioral state (AW: F(2, 20) = 4.09, *p* = 0.034; QW: F(2, 20) = 4.84, *p* = 0.021; NREM: F(2, 20) = 4.66, *p* = 0.023). Specifically, during the 2 h post-injection observation period, clonidine prolonged QW while propranolol prolonged AW and shortened NREM sleep (Supplementary Figure S2). Neither drug affected the average epoch duration in either behavioral state (F(2, 20) ⩽ 2.57, *p* ⩾ 0.10 for all). A mild sedative effect of clonidine was reflected by faster sleep onset (Supplementary Figure S2).

**Figure 3:**
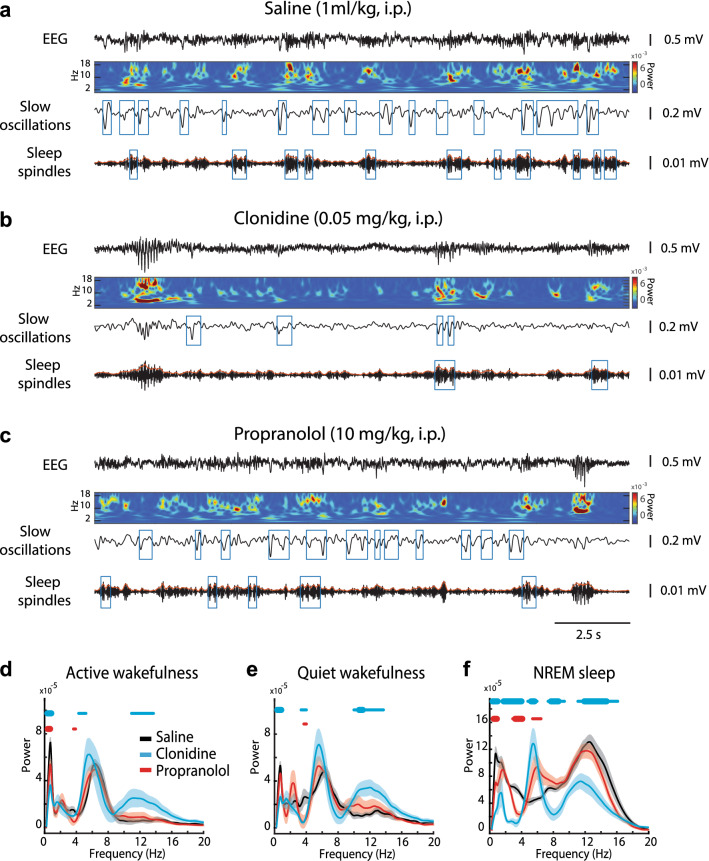
Modulation of the prefrontal EEG by clonidine and propranolol. (**a**–**c**) The representative EEG (0.1–1 kHz) segments are shown for different drug conditions with corresponding time–frequency spectrograms (0.3–20 Hz) and filtered traces used for detection of slow oscillations (SO, 0.3–4 Hz) and sleep spindles (10–16 Hz). Blue rectangles show detected events. Note the clonidine-induced decrease in SO and spindle occurrence. (**d**–**f**) The EEG power spectrum density is plotted for each experimental condition and each behavioral state. The Irregular-Resampling Auto-Spectral Analysis (IRASA) procedure (see Methods) was used to compute the difference between the original power spectrum and the fractal spectrum. Horizontal blue and red lines represent significant differences in the oscillatory component of the EEG power spectrum compared to the saline group. Thin lines: *p* < .05 and thick lines: *p* < .001 for pairwise comparison (independent sample t-test).

**Figure 4:**
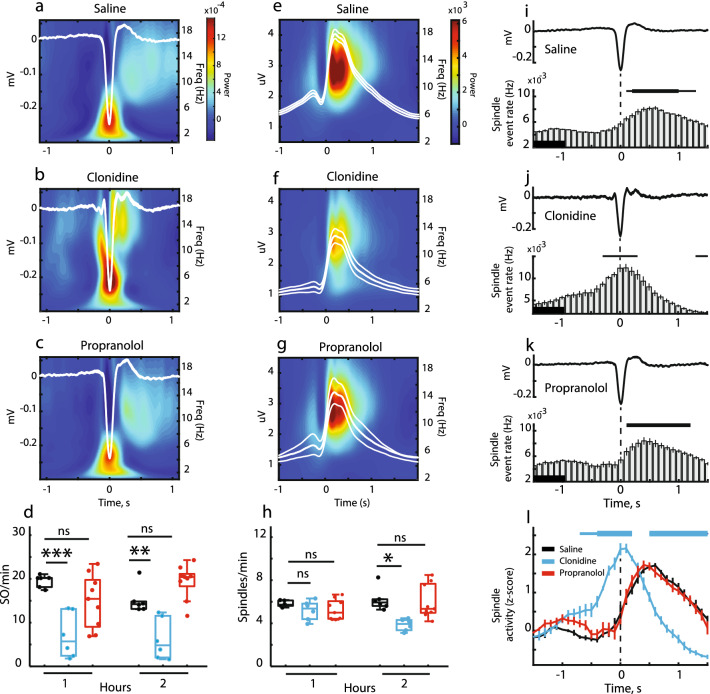
Modulation of the prefrontal EEG oscillations by clonidine and propranolol. (**a**–**c**) The group-averaged peri-event EEG spectrogram (0.1–20 Hz) is plotted around the negative peak of slow oscillation (SO) for saline (**a**), clonidine (**b**), and propranolol (**c**) conditions. The overlay shows the EEG wave average. (**d**) The SO rate during NREM sleep 1 h and 2 h post-injection for saline (black), clonidine (blue) and propranolol (red) groups. The repeated-measures ANOVA confirmed a significant time x drug interaction (F(2, 18) = 5.83, *p* = 0.011) due to decreased SO rate in clonidine-treated rats. (**e**–**g**) The group-averaged peri-event EEG spectrogram (2–20 Hz) is plotted around the spindle onset for different groups. The overlay shows the root mean square of the band-pass (10–16 Hz) filtered EEG. (**h**) The spindle rate during NREM sleep 1 h and 2 h postinjection for saline (black), clonidine (blue) and propranolol (red) groups. There was a significant effect of the drug treatment (F(2, 18) = 9.30, *p* = 0.002) and drug × time interaction (F(2, 18) = 3.14, *p* = 0.067) due to significant decrease in the spindle rate 2 h post-injection in clonidine-treated rats. (**i**–**l**) Temporal coupling between SO and sleep spindles. (**i**–**k**) The EEG wave average (top) and the spindle density (bottom) around the SO negative peak (t = 0, vertical dashed line) are shown for different groups. Bin size: 100 ms. Horizontal lines above the bar plots indicate the epochs with significant differences in spindle density (thin lines: *p* < .01, thick lines: *p* < .001; each bin values were compared to a reference value extracted from a baseline interval [− 1.5 s to − 1.0 s] indicated by a black bar. l, The group averages of z-score normalized spindle density. Horizontal blue lines indicate intervals with significant differences between clonidine and saline groups (thin: *p* < .01; thick: *p* < .001; paired-samples *t* test). Box-whisker plots show the median, the 1st and 3rd quartiles, min/max, and the outliers; dots show individual datapoints. **p < .01, ***p < .001; for pairwise comparisons (Bonferroni post-hoc test).The data after the first drug treatment are shown.

Furthermore, the prefrontal EEG was affected in a drug-specific manner (Fig. 3a–c). Specifically, clonidine increased the sigma (10–16 Hz) power during AW and QW while reducing it during NREM sleep (Fig. 3d–f). Besides, during NREM sleep in drug-treated rats, the delta (< 2 Hz) power was decreased and the power within the 5–7 Hz frequency range was increased due to the appearance of HVS (Fig. 3f). The observed changes in the EEG power spectrum reflected drug-induced modulation of the oscillatory events like SO and sleep spindles (Figs. 3a–c and 4). In clonidine-treated rats, the SO were less frequent (Table 1) and of smaller amplitude (Supplementary Figure S3). There was no effect on the SO (half-wave) duration (F(2, 20) = 2.55, *p* = 0.11). The effect of clonidine on SO was almost immediate and lasted at least for 2 h (Fig. 4d). In clonidine-treated rats, the spindles were more frequent during AW and QW, while their occurrence was decreased during NREM sleep (Table 1). Notably, the effect of clonidine was delayed (Fig. 4h). The spindle power was significantly decreased by clonidine (Supplementary Figure S3). No change was detected in the spindle length (F(2, 20) = 0.08, *p* = 0.92) or the intra-spindle frequency (F(2, 20) = 1.09, *p* = 0.36). The clonidine-induced effects persisted across repeated drug treatments (Supplementary Figure S3). The SO and sleep spindles were unaffected by propranolol (Fig. 4d,h, and Supplementary Figure S3).

We next assessed the drug effect on the temporal coupling between SO and sleep spindles, a well-established phenomenon that has been suggested as a mechanism of the network coordination underlying offline consolidation^42–44^. To this end, we selected NREM sleep episodes and extracted spindles occurring within ± 1.5 s around the SO negative peak. Overall, about 1/3 of spindles was SO-coupled (F(2, 20) = 1.03, *p* = 0.38); however, the spindle grouping around the SO negative peak differed between groups (Fig. 4i–l). In saline- and propranolol-treated rats, the spindles preferentially occurred along the ascending phase of SO reaching the maximum ~ 400 ms after the SO negative peak while in clonidine-treated rats, the spindles grouped around the SO negative peak (Fig. 4l).

Finally, clonidine administration promoted HVS, high-amplitude transient oscillations that rarely occur in drug-free conditions (Table 1 and Supplementary Figure S4). The first HVS appeared as fast as 8.4 ± 1.0 min (range: 5.4 – 11.3 min) after clonidine injection, which was much earlier than after saline (24.6 ± 2.3 min) (range: 16.6 – 30.7 min) or propranolol (27.6 ± 6.2 min) (range: 7.7 – 62.2 min). Remarkably, in 5 out of 6 (83.3%) clonidine-treated rats and 5 out of 9 (55.5%), propranolol-treated rats the HVS were detected during AW state, while the HVS were absent during AW in 5 out of 6 saline-treated rats. In clonidine-treated rats, the HVS rate was higher during the first post-injection hour and this effect remained across days (Supplementary Figure S4). Besides, clonidine increased the HVS duration (Supplementary Figure S4) and decreased the inter-HVS frequency (F(2, 20) = 7.94, *p* = 0.003). The HVS power was unaffected by clonidine (F(2, 20) = 2.28, *p* = 0.13). There were no detectable effects of propranolol on the HVS (Supplementary Figure S4).

## Discussion

In the present study, we showed that repeated administration of clonidine (0.05 mg/kg, i.p.) and propranolol (10 mg/kg, i.p.) after daily exposure to an 8-arm radial maze task impaired spatial memory consolidation in adult male rats. The spatial memory deficiency in drug-treated rats was evident in a higher number of WM and RM errors. Previous studies pointed to a beneficial effect of NREM sleep on WM, possibly due to enhanced SO activity and potentiation of prefrontal–autonomic networks^45,46^. Indeed, parasympathetic activity is increased during NREM sleep and is likely to promote the NE release from the LC via vagal inputs^45^. Thus, coerulear-SO coupling during NREM sleep^35^ might support next-day prefrontal-dependent WM performance^45^. Our results indirectly support this hypothesis as pharmacological suppression of the LC-NE activity during the post-learning period may have indeed affected the prefrontal function that is essential for WM capacity. The lower accuracy of spatial memory that was reflected in RM errors indicated the effect of drugs on the ripple-mediated hippocampal-cortical communication, a key component of the systems-level consolidation^28^. Finally, the strongest memory deficit early in learning indicated the importance of NE transmission for offline processing of newly received information^8–10^.

A lower learning rate in the majority of drug-treated rats was likely related to altered sleep-associated brain activity. Indeed, pharmacological alteration of NE transmission affected the sleep-associated neural dynamics within the thalamocortical and hippocampal networks that are implicated in systems-level memory consolidation^28^. Specifically, both drugs dramatically reduced the occurrence of hippocampal ripples, which are thought to mediate the stabilization of memory-encoding cell assemblies^12,47^. Our results showed that the ripple-generating mechanism requires an optimal level of NE, while both enhanced and decreased LC-NE activity is unfavorable for ripples. Thus, the drug-induced ripple suppression during the post-learning period might explain the spatial memory deficit. This result is consistent with a learning deficit produced by electrical ripple suppression^48,49^. To the best of our knowledge, the noradrenergic effects on the hippocampal ripples have not yet been characterized in behaving animals. Besides its effect on the hippocampal ripples, clonidine, but not propranolol, suppressed SO and sleep spindles, which is consistent with existing literature^50,51^; it also altered SO-spindle coupling.

Although a relatively short (~ 2 h) observation period did not permit characterization of the sleep/wake cycle, the drug effects on the prefrontal EEG were generally consistent with the existing literature^50,51^. We observed drug-specific effects on the sleep/wake pattern and the EEG power spectrum. Namely, clonidine caused slight sedation by accelerating sleep onset and prolonging QW, while propranolol increased AW at the expense of NREM sleep. Both drugs decreased the EEG power within a low (< 2 Hz) frequency range and increased 5–7 Hz power during NREM sleep due to the appearance of the HVSs; clonidine also reduced the sigma (10–16 Hz) power. The effects of clonidine were overall more pronounced, lasted for at least 2 h, and were consistent across daily injections. Of note, the aforementioned effects were observed during the dark (active) phase of the rat circadian cycle, while the drug-induced effects may differ during the light (inactive) phase as well as during the extended post-administration period.

Earlier pharmacological studies have repeatedly demonstrated that modulation of NE transmission after learning affected memory strength^13–15,18,29,52,53^. The higher LC-NE activity during vigilant states has prompted research on its role in online information processing^6,7^, while the role of noradrenergic modulation of the brain networks during low vigilance states, such as sleep, has long remained out of focus. Several studies highlighted the importance of NE acting via β-adrenoreceptors^30,31^ for hippocampal-dependent memory consolidation^10,37,38,54,55^. It is well-established that NE promotes long-term synaptic plasticity^21–25^ that takes place also offline. In our earlier study, we reported that a relative calmness of the LC-NE neurons during NREM sleep was interrupted by transient activity bouts, which were more likely to occur after learning^34^. Optogenetic alteration of the LC activity during NREM sleep impaired the rat's performance on a hippocampus-dependent memory task^37^. Two recent studies by measuring the cortical NE release demonstrated the LC-spindle coupling at the infra-slow temporal scale^26,56^.

Besides the post-learning surges of noradrenergic activity^29,34^, a temporally coordinated engagement of the LC-NE neurons may influence the networks offline. Although the exact mechanism governing the LC activity dynamics during sleep is unknown, temporal relations between the NE level and sleep-associated oscillations exist. The firing rate of LC-NE neurons increases during sleep spindles and declines before the spindle onset^57^. We have earlier demonstrated a temporal coupling between the LC-NE neuron firing and a phase of SO^35^. In our recent study in rats under urethane anesthesia, we showed that phasic LC activation prolonged the cortical state of enhanced excitability and increased the firing rate of prefrontal neurons^36^. We have previously reported that the ripple-triggered electrical LC stimulation produced a spatial memory deficit, likely due to interference with the endogenous LC activity pattern^38^. In our present study, clonidine-induced suppression of NE release affected the spindle generation network that likely disrupted the LC-spindle coupling. Despite the cortical origin of SO, the subcortical inputs from the thalamus^58^ and neuromodulatory nuclei^59^, including the LC^36,60^, affect the SO spatiotemporal dynamics. The NE-mediated modulation of the SO dynamics is further corroborated by the present findings. Specifically, we showed that clonidine suppressed the generation of SO, reduced the SO amplitude, and affected the SO-spindle coupling. The SO-spindle coupling episodes are thought to indicate the reactivation of learning content, with the coupling precision predicting the reactivation strength that is beneficial for memory^61–63^. Thus, a cross-regional temporal coupling, which is a key element of the systems-level consolidation, may require an optimal NE level. The strength of the SO-spindle and spindle-ripple coupling may depend on the firing pattern of LC-NE neurons and the timing of NE release. Temporal and spatial alteration of NE transmission may result in less efficient offline processing and memory deficit. Clearly, there is a pressing need to explore further the functional implications of this finely-tuned temporal relationship between LC-NE neuronal firing and sleep-associated oscillations. Most importantly, the triggers for a coordinated burst of neuromodulatory activity during sleep remain to be determined.

Overall, our results suggest that an optimal level of NE is required for efficient information processing offline. Accumulated experimental evidence and our present findings support the view that NE release during offline states promotes both synaptic and systems-level consolidation^8,11^. The alteration of NE transmission affected essentially all thalamocortical and hippocampal oscillations implicated in systems-level memory consolidation. The ample projections of the LC-NE neurons throughout the brain^64^ provide multiple ways for NE to influence the coordinated activity within memory-supporting networks, including the thalamocortical, prefrontal, and limbic where SO, sleep spindles, and ripples originate^12,65,66^. Future studies shall explore in more detail the noradrenergic mechanisms underlying the emergence, maintenance, and reactivation of a memory-supporting large-scale network.

Despite the different mechanisms of action, both drugs have been shown to reduce LC activity^40,41,67^. Notably, the inhibitory effect of clonidine in vitro was stronger on the hippocampal- versus prefrontal-projecting LC-NE neurons^68^. Clonidine mainly suppresses presynaptic NE release via α2-adrenoreceptors and propranolol blocks NE transmission via β–adrenergic receptors. Besides, propranolol may inhibit NE reuptake, potentiate NE transmission at α1-adrenoreceptors, and decrease dopaminergic transmission due to inhibition of catecholamines synthesis^69^; it may also affect serotonergic transmission due to its affinity to central 5-HT receptors^70^. The convergent effects of two clinically relevant noradrenergic drugs, despite their acting on different adrenoreceptors, suggest that pharmacologically altered NE transmission during the post-learning period may interfere with the memory-supporting network and cause a cognitive side effect of the therapeutic use of these drugs.

## Methods

We used 38 male Sprague–Dawley rats (300 – 400 g, Charles Rivers Laboratories, Sulzfeld, Germany). The animal facility had environmentally controlled conditions: 12 h light/dark cycle (lights off at 8:00 A.M.), 20–23 °C temperature, and 40–60% humidity. Rats were housed in pairs or individually, and had ad libitum access to food and water, except for food restriction (~ 15 g of food pellets per rat per day) during the learning experiment. Rats' weight was controlled daily to ensure that it is not below 85% of the rat's weight at libitum. All experiments were conducted in full compliance with Directive 2010/63/EU of the European Parliament, the German Animal Welfare Act (TierSchG), and the Animal Welfare Laboratory Animal Ordinance (TierSchVersV). The study was reviewed by the regional animal welfare committee according to §15 of the German Animal Welfare Act (Kommission nach §15 des Tierschutzgesetzes), the ethics commission (§15 TierSchG) and approved by the state authority (Regierungspräsidium, Tübingen, Baden-Württemberg, Germany, Referat 35, Veterinärwesen). The present study is performed in accordance with ARRIVE guidelines (https://arriveguidelines.org).

### Surgery and electrophysiological recordings

The surgical and recording procedures have been described in detail elsewhere^38,71^. Briefly, an isoflurane-anesthetized (5% for induction, ~ 2% for maintenance) rat was fixed in a stereotaxic frame (David Kopf Instruments, Tujunga, CA) with a head angle adjusted at zero degrees. A local anesthetic (Lidocard 2%, B. Braun, Melsungen, Germany) was injected subcutaneously before craniotomies for the electrode insertion. For the electroencephalogram (EEG), two stainless-steel screws (0.86 mm diameter) were fixed above the prefrontal cortex and the cerebellum. Two single platinum-iridium electrodes (FHC Inc., Bowdoin, ME) glued together with a 100 to 200 µm distance between the tips were mounted on a homemade movable drive and implanted in the dorsal HPC (AP = -3.5 mm, L = 2.0 mm, DV = 2.0 mm). Online monitoring of neural activity ensured accurate targeting of the CA1 area of the dorsal HPC during the electrode implantation. The movable drive allowed additional adjustment of the electrode depth for optimal ripple recording during maze training. For electromyography (EMG), a silver wire electrode was inserted in the neck muscle. A 3D-printed plastic frame connected to a copper mesh for protection of the electrode wires and isolation from electrical noise was glued to the skull. Body temperature (~ 37 °C), heart rate, and blood oxygenation (above 90%) were monitored. During 5–7 days of post-surgical recovery, rats were treated with an antibiotic (10 mg/kg, i.p., Baytril, Bayer) and painkiller (5 mg/kg, i.p., Rymadil, Zoetis).

After post-surgery recovery rats were habituated to the sleep box (black Plexiglas, 20 × 20 × 80 cm) and a cable connection procedure. The implant was connected to the Neuralynx Digital Lynx acquisition system (Neuralynx, Bozeman, MT) through a 32-channel head stage (Neuralynx, Bozeman, MT) and a custom-made adaptor (SSD-10-SS-GS, Omnetic, Minneapolis, MN). A broadband (0.1 Hz—8 kHz) extracellular signals from EEG and HPC electrodes were digitized at 32 kHz. The electrode placement in the HPC was optimized for ripple detection at least 24 h before the recording started. Each recording session began at the same time for each animal and lasted for ~ 2 h. This observation period was selected based on previous evidence that the early phase of offline consolidation is NE-dependent^8^. The recording was made in dim light and during the active (dark) phase of the circadian rhythm, which is the most optimal for training rats.

### Spatial memory task

The same maze task and training protocol was used as described in detail elsewhere^38^. Rats were trained on an elevated eight-arm radial maze (Fig. 1a). Maze arms (66 cm long × 10 cm wide) with a small pit (1 cm diameter) at the end for food reward (chocolate milk) extended from a central platform (30 cm diameter). Two large visual cues were fixed on the black curtains surrounding the maze. During the inter-trial interval, a rat was placed on an elevated stand. Rat behavior was video-monitored. After habituation and pretraining (*Supplementary Methods*), 3 out of 8 maze arms were baited; reward locations were randomly assigned for each rat and maintained the same for all training sessions (3 trials per day for 7 days). A trial started by placing a rat on the central platform and lasted until all 3 rewards were collected or 5 min elapsed. The maze surface was wiped after each trial to minimize olfactory cues. The following behavioral variables were registered and analyzed: number of maze arms visited, trial time, choice accuracy (calculated as a ratio between the baited and total number of arms visited), working memory (WM) errors (number of re-entries into visited arms), reference memory (RM) errors (entries into non-baited arms).

### Drug injections

The clonidine and propranolol powder (Sigma Aldrich, Burlington, MA) was dissolved in saline and injected intraperitoneally (i.p.) in a volume of 1 ml/kg immediately after each maze session (clonidine: 0.05 mg/kg, i.p.; propranolol: 10 mg/kg, i.p.). Control rats received saline (1 ml/kg, i.p.). Clonidine suppresses NE transmission^72^ and in a dose of 0.05 mg/kg (i.p.) has been shown in rats to reduce the LC activity to ~ 60% baseline level^67^. Propranolol, a β-adrenoreceptor antagonist, after repeated treatment at a moderate dose (< 40 mg/kg) inhibits the LC activity^40,41^. Besides, propranolol in a dose of 10 mg/kg (i.p.) was shown to modulate NE-mediated excitability of the hippocampal neurons^73^ while it did not affect rat spontaneous behavior^74–76^.

The half-life of clonidine may greatly vary between 6 and 23 h depending on the dosage, chronic use, and metabolism level^75,77^. Typically, the plasma peak levels are reached 60–90 min after clonidine administration. The plasma half-life of propranolol is 3 to 6 h with a peak 1–3 h after ingestion^76^. The elimination half-life of propranolol is approximately 8 h (https://pubchem.ncbi.nlm.nih.gov/compound/Propranolol). This, according to the pharmacokinetics of both drugs, the drug concentration was close to the highest during the observation period of up to 2 h post-injection. Importantly, both drugs are effectively washed out within less than 24 h.

### Perfusion and histology

Rats were euthanized (100 mg/kg i.p., Narcoren, Merial), and perfused transcardially with 0.9% saline followed by 4% paraformaldehyde. Brains were sliced (50 µm) using a horizontal freezing microtome (Microm HM 440E, Thermo Fischer Scientific, Waltham, MA). To verify the electrode placement, Nissl-stained brain sections were examined under the microscope (Axiovision, Zeiss, Oberkochen, Germany).

### Event detection

The SO, sleep spindles, and hippocampal ripples were detected as previously described elsewhere^71,78^. The EEG signal was downsampled to 1 kHz and band-pass (0.3 – 4 Hz) filtered. For SO detection, zero crossings of filtered EEG occurring within a 0.4 to 1-s time window were extracted. Two consecutive negative‐to‐positive zero-crossings with a negative peak amplitude below − 100 μV and negative-to-positive peak-to-peak amplitude exceeding 120 μV were assigned as SO. For spindle detection, band-pass (10–16 Hz) filtered EEG was rectified and smoothed. The spindles were detected by a signal amplitude exceeding a threshold of 1.5 standard deviations (SD) of the mean signal during NREM sleep epochs for 0.4—2.0 s. Spindles occurring within less than 50 ms were fused. The HVSs were detected using the same algorithm with adjusted parameters (band-pass: 5–7 Hz, threshold: 2.5 SD, min/max: 0.7 s/3 s). Two HVSs occurring in less than 200 ms were fused. To prevent double detection, sleep spindles were identified on HVS-free epochs. For ripple detection, HPC LFP was band-pass (120–250 Hz) filtered, rectified, and smoothed. The ripples were detected by a signal amplitude exceeding a threshold of 3 to 5 SDs for 0.025 to 0.5 s. For all events, on- and offsets were defined as ascending and descending zero-crossings, respectively, using a 1 SD threshold. The oscillatory power was calculated as the integral of the envelope of the Hilbert‐transformed signal between the event on- and offset. The SO-spindle temporal coupling was assessed as described elsewhere^43^. The SO negative peak was used as a reference event (t = 0), the peaks and troughs of spindles occurring within ± 1.5 s were extracted and the event-correlation histogram was generated. The bin count was converted to the event rate, z-transformed, and group averaged.

### Sleep scoring and EEG power spectral analysis

The sleep scoring was based on visual-assisted classification for consecutive 10-s epochs of the EEG and EMG signals^79^. The artifact-free EEG was classified into active wakefulness (AW, low-amplitude fast EEG, and high EMG), quiet wakefulness (QW, low-amplitude fast EEG, and low EMG), and NREM (high-amplitude slow EEG and low EMG). The REM sleep epochs during 2 h-recording were rare and not analyzed. The EEG power spectra were calculated for each behavioral state using Matlab (Mathworks, USA) and the FieldTrip toolbox^80^. The EEG signal was segmented into 3-s epochs with a 0.5-s overlap. The Fast Fourier Transformation was applied to Hanning tapered data segments to calculate the single-sided amplitude spectrum. To separate the fractal and oscillatory components in the EEG power spectrum, we used the Irregular-Resampling Auto-Spectral Analysis procedure^81^. The time–frequency plots were generated using a sliding Hanning tapered window and a frequency-dependent length of 3 cycles. The frequency range of 0.05 to 20 Hz (0.1 Hz step) was used for SO, sleep spindles, and HVS, and 50 to 300 Hz (0.05 Hz step and 9 cycles) for the hippocampal ripples.

### Experimental design and statistical analysis

Thirty-two rats were trained on the maze, of those 19 rats were implanted with electrodes, and treated either with clonidine (n = 16), propranolol (n = 11), or saline (n = 14) after each learning session. Additional 9 rats were implanted with electrodes and used for electrophysiology only. The behavioral variables were session-averaged and submitted to the repeated-measures analysis of variance (ANOVA) with the learning session as a repeated factor and the drug treatment as a group factor. The Greenhouse–Geisser correction was applied when the sphericity assumption was violated. The K-means cluster analysis was used to reveal the between-subject variability in the learning rate. Specifically, we submitted the choice accuracy (as defined in *Spatial memory task* Method section) for all learning sessions and split the time series into two clusters differing by the learning dynamics. We then calculated the proportion of rats in each cluster for each experimental group. The variables derived from electrophysiological signals such as sleep onset, epoch duration, event rate, etc., were submitted to a one-way ANOVA with the drug treatment as a group factor followed, when appropriate, by the Bonferroni post-hoc tests. The post-injection event rate was compared using the repeated measures ANOVA with a 1 h-time window and across repeated drug treatments. The paired-sample t-test was used to reveal the between-group difference in the peri-event histograms. The statistical significance (α-value) was set at *p* = 0.05. IBM SPSS Statistics (v.22) was used for statistical analysis. The Matlab (R2014a) and Adobe Illustrator® software packages were used for the visualization of results.

## Supplementary Information

Supplementary Information.

## Data Availability

The datasets used and/or analysed during the current study are available from the corresponding author upon reasonable request.
