# Supplementary material for: Altered norepinephrine transmission after spatial learning impairs sleep-mediated memory consolidation in rats
Source: Sci Rep. 2023 Mar 14;13:4231. doi: 10.1038/s41598-023-31308-1 (PMC10014950; doi:10.1038/s41598-023-31308-1)
Supplement: Supplementary file 1 — Supplementary Information. [file 41598_2023_31308_MOESM1_ESM.docx]

# Supplementary Methods

*Spatial memory task*

A custom-made eight-arm radial maze was fixed 84 cm above the floor and surrounded by black curtains. Eight alleys (66 cm long x 10 cm wide) extended from a central round platform (30 cm diameter). A small (1 cm diameter) round pit was located at the end of each maze alley for food reward (a few drops of chocolate milk, Fig. 1A). Two large visual cues were hung on the curtains surrounding the maze. An elevated box stand (~80 cm above the floor) for keeping a rat in the transport cage during the inter-trial interval (ITI) was located at a fixed corner of the room and also served as an extra-maze cue. The experimental room was dimly lit. After placing a rat on the maze, the experimenter quickly left the room and monitored rat behavior through a video camera until the trial ended. Rat training took place during the dark phase of the light/dark cycle and at the same time for each rat.

Before training on the maze, rats were familiarized with the chocolate milk in their home cages and habituated to gentle handling by an experimenter. During habituation trials (typically, 2 sessions), drops of chocolate milk were distributed over the entire maze surface, and a food-deprived rat was placed on the central platform and was allowed to collect the reward for 20 to 30 minutes. In case of any signs of stress (e.g. freezing), a rat was picked up from the maze, gently handled by the experimenter, and placed on the inter-trial stand for a few minutes to calm down. Once a rat was readily drinking chocolate milk, the drops of chocolate milk were gradually distributed closer to the reward pits at the end of each maze alley. The habituation phase ended when a rat placed on the central platform readily retrieved a reward at the end of any maze alley. During pre-training sessions, chocolate milk was distributed only in the reward pits at the end of each maze alley. A rat was placed on the central platform and allowed to explore the maze until 3 rewards were collected or 5 min had elapsed. The same trial was repeated 3 times with an ITI of ~1 min. During ITI, the maze surface was wiped to remove olfactory cues and reward pits re-baited. The elevated box stand was used for keeping a rat during ITI. The pre-training phase was completed when a rat placed on the central platform collected 3 rewards within 5 min.

During learning sessions, three out of eight arms were baited. The position of rewarded pits was randomly assigned for each rat and maintained fixed for eight daily sessions. This task design while minimizing working memory load required spatial (reference) memory for successful performance. Each training session consisted of 3 trials. In each trial, a rat was placed on the central platform and allowed 5 min to collect rewards. When the trial ended, a rat was returned to the transport cage for ITI on an elevated box stand, the maze surface was wiped, and reward pits re-baited.

In each trial, the following behavioral variables were registered: 1) a total number of maze arms visited; an entry to the maze arm was considered when a rat left the central platform with all four limbs. 2) trial time, the time required to collect 3 rewards; in case 3 rewards were not collected within 5 min, the cut-off time of 5-min was assigned as trial time, 3) accuracy of task performance, accuracy was calculated as the ratio between the baited arms visited and the total number of arms visited 4) working memory errors (WM errors) were calculated as a number of re-entries into visited arms, 5) reference memory errors (RM errors) were calculated as a number of entries into non-baited arms using the following formula: RM errors = Total arms visited – (Baited arms visited + WM errors).

Supplementary Table S1. Experimental conditions for each rat and data inclusion/exclusion.

|  | **Rat ID** | **Drug** | **Exp. Condition** | **Maze** | **EEG** | **HPC** | **Notes** |
| --- | --- | --- | --- | --- | --- | --- | --- |
| 1 | 85.2 | Clonidine | Maze/Pharm/Ephys | Trained | EEG | CA1 dHPC |  |
| 2 | 87.2 | Clonidine | Maze/Pharm/Ephys | Trained | EEG | CA1 dHPC |  |
| 3 | 91.1 | Clonidine | Maze/Pharm/Ephys | Trained | EEG | CA1 dHPC |  |
| 4 | 95.2 | Clonidine | Maze/Pharm/Ephys | Trained | EEG | CA1 dHPC |  |
| 5 | 87.3 | Clonidine | Maze/Pharm/Ephys | Trained | EEG | no ripples |  |
| 6 | 113.2 | Clonidine | Maze/Pharm/Ephys | Trained | EEG | no ripples |  |
| 7 | 1025.1 | Clonidine | Maze/Pharm/Ephys | Trained | no EEG | no ripples | unreliable EEG |
| 8 | 70.2 | Clonidine | Maze/Pharm/Ephys | Trained | no EEG | no ripples | unreliable EEG |
| 9 | 71.2 | Clonidine | Maze/Pharm/Ephys | Trained | no EEG | no ripples | unreliable EEG |
| 10 | 127.3 | Clonidine | Maze/Pharm/----- | Trained | n/a | n/a |  |
| 11 | 161.1 | Clonidine | Maze/Pharm/----- | Trained | n/a | n/a |  |
| 12 | 161.2 | Clonidine | Maze/Pharm/----- | Trained | n/a | n/a |  |
| 13 | 646.2* | Clonidine | -----/Pharm/Ephys | n/a | n/a | CA1 dHPC | Ripple, additional |
| 14 | 673.1* | Clonidine | -----/Pharm/Ephys | n/a | n/a | CA1 dHPC | Ripple, additional |
| 15 | 711.2* | Clonidine | -----/Pharm/Ephys | n/a | n/a | CA1 dHPC | Ripple, additional |
| 16 | 814.1* | Clonidine | -----/Pharm/Ephys | n/a | n/a | CA1 dHPC | Ripple, additional |
|  |  |  |  | **Maze (n=12)** | **EEG (n=6)** | **Ripple (n=8)** |  |
| 17 | 131.1 | Propranolol | Maze/Pharm/Ephys | Trained | EEG | CA1 dHPC |  |
| 18 | 131.2 | Propranolol | Maze/Pharm/Ephys | Trained | EEG | CA1 dHPC |  |
| 19 | 132.2 | Propranolol | Maze/Pharm/Ephys | Trained | EEG | CA1 dHPC |  |
| 20 | 132.3 | Propranolol | Maze/Pharm/Ephys | Trained | EEG | CA1 dHPC |  |
| 21 | 140.2 | Propranolol | Maze/Pharm/Ephys | Trained | EEG | CA1 dHPC |  |
| 22 | 140.3 | Propranolol | Maze/Pharm/Ephys | Trained | EEG | CA1 dHPC |  |
| 23 | 140.4 | Propranolol | Maze/Pharm/Ephys | Trained | EEG | CA1 dHPC |  |
| 24 | 131.3 | Propranolol | Maze/Pharm/Ephys | Trained | no EEG | no ripples | unreliable EEG |
| 25 | 131.4 | Propranolol | Maze/Pharm/Ephys | Trained | EEG | no ripples |  |
| 26 | 140.1 | Propranolol | Maze/Pharm/Ephys | Trained | EEG | no ripples |  |
| 27 | 161.4 | Propranolol | Maze/Pharm/----- | Trained | n/a | n/a |  |
| --- | 711.2* | Propranolol | ------/Pharm/Ephys | n/a | n/a | CA1 dHPC | Ripple, additional |
| --- | 814.1* | Propranolol | ------/Pharm/Ephys | n/a | n/a | CA1 dHPC | Ripple, additional |
|  |  |  |  | **Maze (n=11)** | **EEG (n=9)** | **Ripple (n=9)** |  |
| 28 | 91.2 | Saline | Maze/Pharm/Ephys | Trained | EEG | no ripples |  |
| 29 | 95.1 | Saline | Maze/Pharm/Ephys | Trained | EEG | no ripples |  |
| 30 | 98.1 | Saline | Maze/Pharm/Ephys | Trained | EEG | no ripples |  |
| 31 | 98.2 | Saline | Maze/Pharm/Ephys | Trained | EEG | CA1 dHPC |  |
| 32 | 113.1 | Saline | Maze/Pharm/Ephys | Trained | EEG | no ripples |  |
| 33 | 107.1 | Saline | Maze/Pharm/Ephys | Trained | no EEG | no ripples | unreliable EEG |
| 34 | 107.2 | Saline | Maze/Pharm/Ephys | Trained | EEG | no ripples |  |
| 35 | 127.1 | Saline | Maze/Pharm/----- | Trained | n/a | n/a |  |
| 36 | 127.2 | Saline | Maze/Pharm/----- | Trained | n/a | n/a |  |
| --- | 646.2* | Saline | -----/Pharm/Ephys | n/a | n/a | CA1 dHPC | Ripple, additional |
| --- | 673.1* | Saline | -----/Pharm/Ephys | n/a | n/a | CA1 dHPC | Ripple, additional |
| 37 | 673.2 | Saline | -----/Pharm/Ephys | n/a | n/a | CA1 dHPC | Ripple, additional |
| --- | 711.2* | Saline | -----/Pharm/Ephys | n/a | n/a | CA1 dHPC | Ripple, additional |
| --- | 814.1* | Saline | -----/Pharm/Ephys | n/a | n/a | CA1 dHPC | Ripple, additional |
| 38 | 852.2 | Saline | -----/Pharm/Ephys | n/a | n/a | CA1 dHPC | Ripple, additional |
|  |  |  |  | **Maze (n=9)** | **EEG (n=6)** | **Ripple (n=7)** |  |

* - multiple drug conditions tested

Supplementary Table S2. Behavioral variables on the first learning session.

|  | *Trial time, s* | *Rewards, n* | *Arm entries, n* | *Accuracy, a.u.* |
| --- | --- | --- | --- | --- |
| *Saline*  *(n = 9)* | 208.3 ± 20.2 (114.3/300.0) | 2.6 ± 0.2 (1.3/3.0) | 7.0 ± 0.7 (4.0/11.0) | 41.1 ± 2.8 (26.0/51.0) |
| *Clonidine*  *(n = 12)* | 214.8 ± 23.1 (91.0/300.0) | 2.5 ± 0.2 (1.3/3.0) | 6.3 ± 0.4 (4.3/8.3) | 44.3 ± 4.9 (20.6/75.0) |
| *Propranolol*  *(n = 11)* | 169.7 ± 22.2 (85.7/300.0) | 2.8 ± 0.1 (1.7/3.0) | 8.2 ± 0.6 (6.0/12.0) | 39.5 ± 2.6 (22.5/51.0) |

Means ± S.E.M. and the range (min/max) for each variable are shown.

Note, no significant between-group difference (one-way ANOVA, all p-values > 0.05).


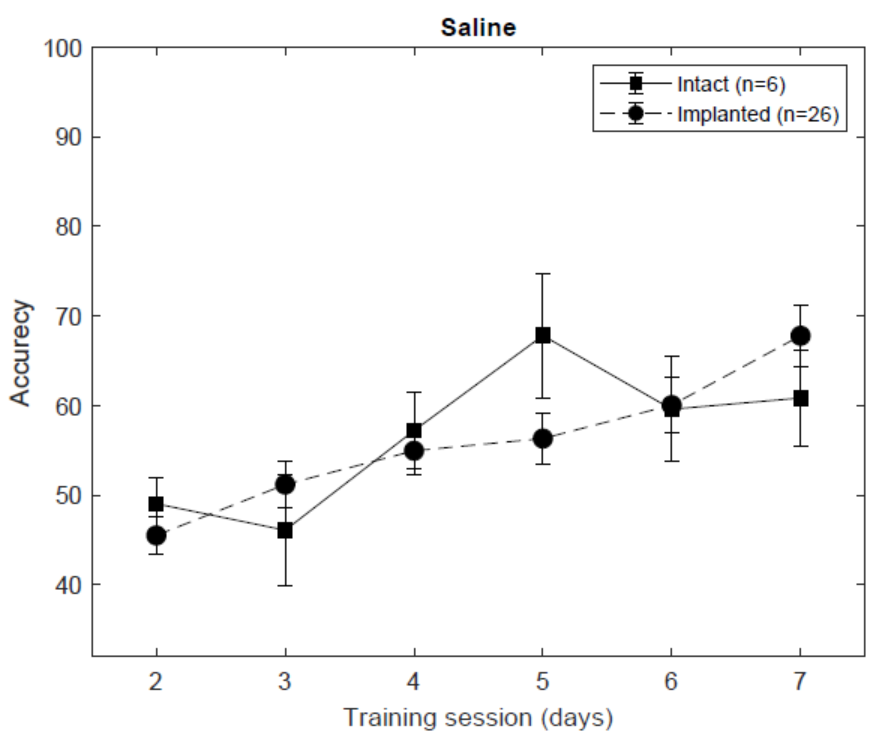


**Supplementary Figure S1. The implantation of chronic electrodes did not affect spatial learning.**


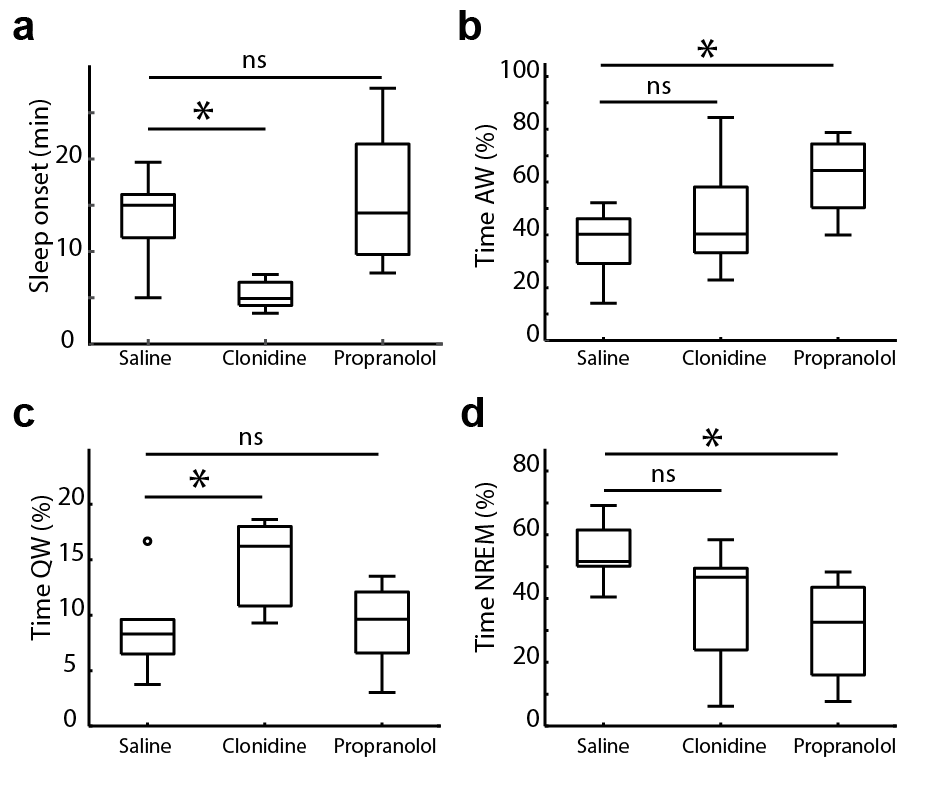


**Supplementary Figure S2. The effects of clonidine (0.05 mg/kg, i.p.) and propranolol (10 mg/kg, i.p.) on the sleep/awake pattern.** The sleep onset latency **(a)** and time spent in each behavioral state (**b-d**) are shown for saline (n = 6), clonidine (n = 6) and propranolol (n = 9). AW - active wakefulness, QW - quiet wakefulness, NREM – non-rapid eye movement sleep. Box-whisker plots show the median, the 1st and 3rd quartiles, min/max, and the outliers. *p < .05, **p < .01 (Bonferroni post-hoc test).


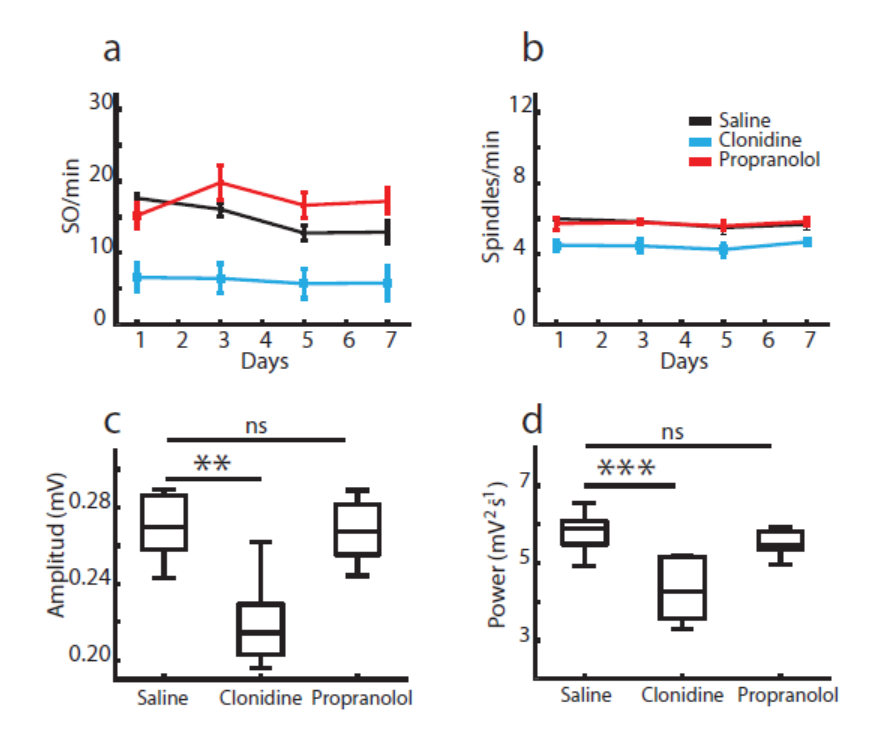


**Supplementary Figure S3. The effects of clonidine (0.05 mg/kg, i.p.) and propranolol (10 mg/kg, i.p.) on the sleep-associated oscillations. a,b,** The event occurrence rate across repeated drug treatments. The repeated-measures ANOVA confirmed a consistently decreased SO **(a)** and spindle **(b)** rate after clonidine (SO: F(2, 18) = 14.79, p = 0.000; day x drug interaction: F(3.7, 33.3) = 1.76, p = 0.17; spindle: F(2, 18) = 12.02, p = 0.000, day x drug interaction: F(6, 34) = 0.29, p = 0.94). The event rate is averaged over 2h post-injection. **c,** The SO amplitude for different groups. The number of SOs was matched across rats and experimental conditions. When necessary, the events were extracted from more than one session (saline: 1415.3 ± 82.0 SO/rat, n = 6 rats; clonidine: 1098.2 ± 140.2 SO/rat, n = 6 rats; propranolol: 1121.1 ± 100.0 SO/rat, n = 9 rats). **d,** The sleep spindle power for different groups. The dataset comprised 506.0 ± 35.9 spindles/rat for saline (n = 6 rats), 461.3 ± 38.5 spindles/rat for clonidine (n = 6 rats), and 449.9 ± 35.7 spindles/rat for propranolol (n = 9 rats). Box-whisker plots show the median, the 1st and 3rd quartiles, min/max, and the outliers. **p < .01, ***p < .001 (Bonferroni post-hoc test).


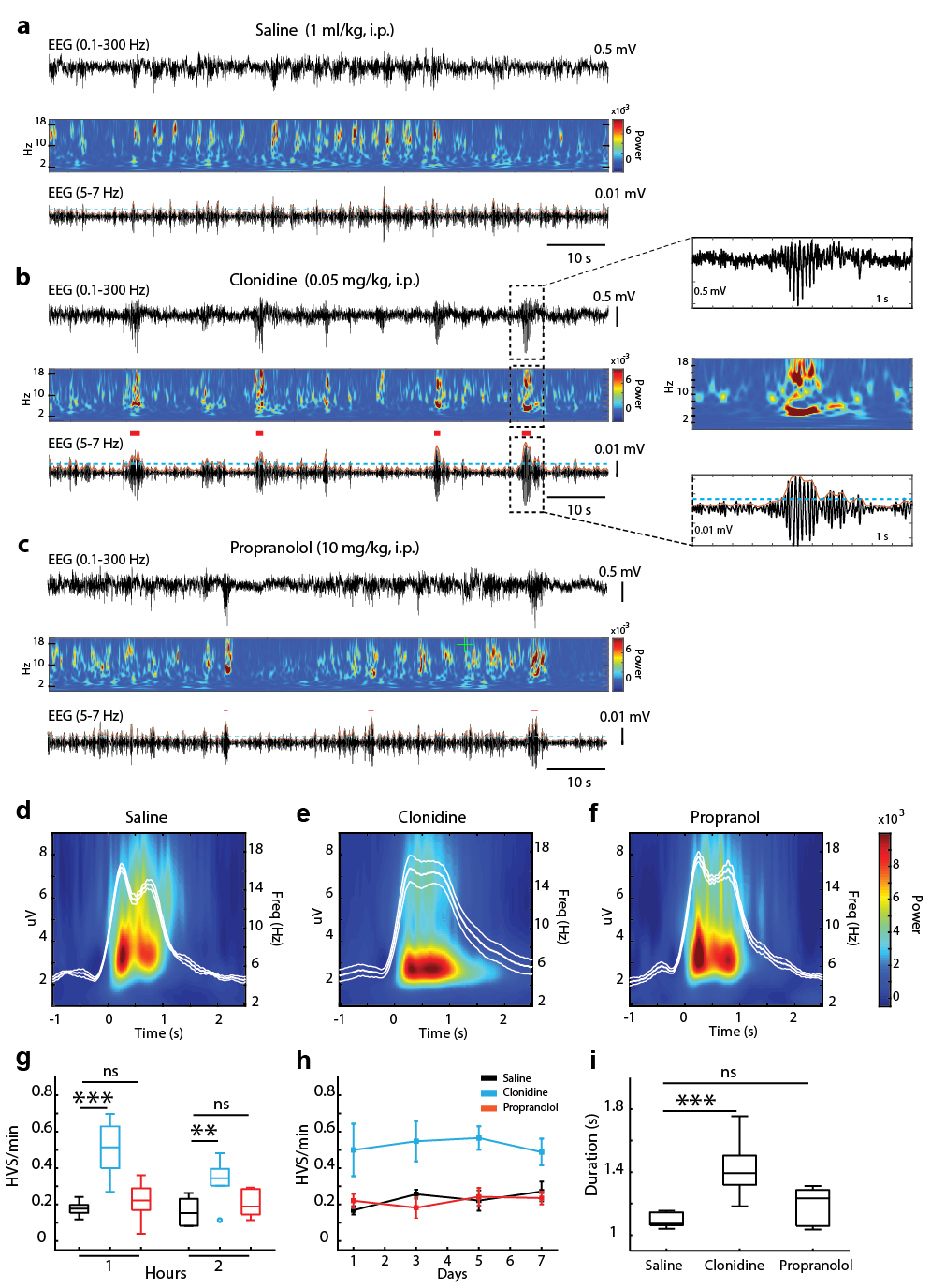


**Supplementary Figure S4. Clonidine promotes the high-voltage spindles (HVS). a-c,** Representative EEG segments are shown for different drug conditions with corresponding time-frequency spectrograms (0.3 - 20 Hz) and filtered traces used for detection of the high voltage spike-and-wave patterns (HVS, 5-7 Hz). Horizontal blue line shows the detection threshold. Red marks show detected HVS. **d-f,** The group-averaged peri-event EEG power spectrum (2–20 Hz) around the HVS onset is shown for different groups. The overlay shows the root mean square (RMS) of the band-pass (5-7 Hz) filtered EEG. **g,** The HVS rate during NREM sleep 1h and 2h post-injection. **h,** The HVS rate across repeated drug treatments. The data for the 1^st^ post-injection hour, when the strongest effect was observed, are shown. The repeated measured ANOVA revealed significant effect of the drug treatment (F(2, 18) = 16.5, p =0.0008) with no day x drug interaction (F(4.2 ,37.8) = 0.4, p = 0.78). **i,** The HVS duration. The dataset contained 42.0 ± 4.1 HVS/rat for saline (n = 6 rats), 171.3 ± 42.8 HVS/rat for clonidine (n = 6 rats), and 30.1 ± 3.9 HVS/rat for propranolol (n = 9 rats). The lowest count of HVS in the propranolol group was due to the shorter duration of NREM sleep when the HVS predominantly occurs. Box-whisker plots show the median, the 1st and 3rd quartiles, min/max, and the outliers. **p < .01, ***p < .001 (Bonferroni post-hoc test).
